# The use and misuse of oxytocin: a study in rural Karnataka, India

**DOI:** 10.1186/1753-6561-6-S1-P12

**Published:** 2012-01-16

**Authors:** Baneen Karachiwala, Zoe Matthews, Asha Kilaru

**Affiliations:** 1 Belaku Trust, Bangalore, India; 2 Department of social sciences, University of Southampton, UK

## Introduction

Poor quality of care remains key constraint to safe motherhood especially for women from poor families in a context of rapid expansion of maternity healthcare services. In India, deliveries at healthcare institutions have increased in last three years.

There is evidence that oxytocin is a drug is often misused to speed labours (childbirth) in overcrowded labour wards or even in home settings. In India this phenomenon has not been extensively studied, especially since the expansion of institutional deliveries. To order to study use of oxytocin, we collected information on drugs administered for labour augmentation as part of a prospective study of pregnancy and childbirth in south India.

## Methods

We randomly selected 39 villages across 13 primary health centres in a rural block of Ramnagar district in Karnataka. Subsequently we purposively selected 41 villages located adjacent to villages selected earlier to meet enrolment target. All women who planned to deliver within study area and were in the third trimester of pregnancy were enrolled (642) during 2007-2009.

Of the total number of respondents, 501 women delivered at healthcare institutions and 99 at home. We conducted in-depth interviews with healthcare providers.

## Results

Healthcare providers revealed that oxytocin was the most commonly available and used drug for labour augmentation. Of the women who delivered child at home, 76.4% were administered oxytocin mainly by an Auxiliary Nurse Midwife (ANM) in the dosage ranging between one to five injections. Most of these women reported that these intramuscularly administered injections were “to increase labour”. Of the women who gave delivered child at healthcare institutions, 23% were administered oxytocin – mainly via intramuscular injection.

Many women reported that healthcare providers did not remain present after administration of oxytocin. Also, the cost of procuring the oxytocin injections were reported high in spite of subsidised rates.

## Discussion

We believe that our study findings would be a conservative estimate of the extent of oxytocin use since many women might have received the drug without explanation suggesting unnecessary use in government as well as private healthcare services.

We recommend need to ensure compliance with clinical protocols and guidelines for the safe use of oxytocin, a drug critical to safe motherhood but with considerable scope for misuse.

